# Improvement of Metal-Doped β-TCP Scaffolds for Active Bone Substitutes via Ultra-Short Laser Structuring

**DOI:** 10.3390/bioengineering10121392

**Published:** 2023-12-06

**Authors:** Íris Soares, Lamborghini Sotelo, Ina Erceg, Florian Jean, Marie Lasgorceix, Anne Leriche, Maja Dutour Sikirić, Katarina Marušić, Silke Christiansen, Albena Daskalova

**Affiliations:** 1 Laboratory of Micro and Nano-Photonics, Institute of Electronics, Bulgarian Academy of Sciences, 72 Tsarigradsko Chaussee Blvd, 1784 Sofia, Bulgaria; 2 Institute for Nanotechnology and Correlative Microscopy vV INAM, Äußere Nürnberger Str. 62, 91301 Forcheim, Germany; lamborghini.sotelo@inam-forchheim.de (L.S.); silke.christiansen@ikts.fraunhofer.de (S.C.); 3 Friedrich-Alexander University Erlangen-Nürnberg, Staudstraße 7, 91058 Erlangen, Germany; 4 Fraunhofer Institute for Ceramic Technologies and Systems IKTS, Äußere Nürnberger Str. 62, 91301 Forcheim, Germany; ina.erceg@ikts.fraunhofer.de; 5 University Polytechnique Hauts-de-France, INSA Hauts-de-France, CERAMATHS—Laboratoire de Matériaux Céramiques et de Mathématiques, F-59313 Valenciennes, France; florian.jean@uphf.fr (F.J.); marie.lasgorceix@uphf.fr (M.L.); anne.leriche@uphf.fr (A.L.); 6 Laboratory for Biocolloids and Surface Chemistry, Division of Physical Chemistry, Ruđer Bošković Institute, Bijenička cesta 54, 10000 Zagreb, Croatia; maja.sikiric@irb.hr; 7 Radiation Chemistry and Dosimetry Laboratory, Division of Materials Chemistry, Ruđer Bošković Institute, Bijenička cesta 54, 10000 Zagreb, Croatia; kmarusic@irb.hr; 8 Frei Iniverssität Berlin, Arnimalle 14, 14995 Berlin, Germany

**Keywords:** bone substitute, β-TCP, laser ablation, femtosecond laser

## Abstract

Various efforts have been made to develop antibacterial biomaterials capable of also sustaining bone remodulation to be used as bone substitutes and reduce patient infection rates and related costs. In this work, beta-tricalcium phosphate (β-TCP) was chosen due to its known biocompatibility and use as a bone substitute. Metal dopants were incorporated into the crystal structure of the β-TCP, and disks were produced from this material. Magnesium and strontium, as well as copper and silver, were chosen as dopants to improve the osteogenic and antibacterial properties, respectively. The surface of the β-TCP samples was further modified using a femtosecond laser system. Grid and line patterns were produced on the plates’ surface via laser ablation, creating grooves with depths lower than 20 μm and widths between 20 and 40 μm. Raman and FTIR analysis confirmed that laser ablation did not result in the degradation or phase change of the materials, making it suitable for surface patterning. Laser ablation resulted in increased hydrophilicity of the materials, as the control samples (non-ablated samples) have WCA values ranging from 70° to 93° and become, upon laser ablation, superwicking surfaces. Confocal measurements show an increase in specific surface area of 50% to 200% compared to the control. Overall, the results indicate the potential of laser ablation to improve the surface characteristics of β-TCP, which may lead to an improvement in the antibacterial and osteogenic properties of the produced materials.

## 1. Introduction

In recent years, bone grafts have become the second most commonly transplanted tissue, with over 2.2 million grafts performed in traumatology, oncology, or orthopedics worldwide. Several bone replacements are currently available, such as an autograph, xenograft, and allograph. The first two consist of bone from the patient and external sources, respectively, while allographs comprise bone mimicking calcium phosphates such as hydroxyapatite (HA) and tri-calcium phosphates (TCP) [1]. Autografts are the gold standard of bone grafting since they do not cause implant rejection but require two surgeries, and only a limited amount of bone can be transplanted without compromising the donor site [2,3]. Allografts and xenografts are limited by availability and tissue matching and may result in disease transmission and implant failure. In fact, 30–60% of these implants fail within the first 10 years after implantation [3].

Due to these issues, tissue engineering and regenerative medicine have focused on alternatives to these substitutes. Different bone substitutes have been developed, differing in composition, application, and characteristics. Despite these differences, bone substitutes always require biocompatibility, bio-resorbability, structural and mechanical similarity to bone characteristics, and, if possible, promotion of osteoconductivity and osteoinductivity. In response to these demands, calcium phosphate ceramics, specifically HA and β-tricalcium phosphate (β-TCP), which are chemically similar to the bone mineral component, have been widely used since the 1980s [4]. These materials differ in their resorption rates. HA is almost insoluble and therefore is not replaced with newly formed tissue, while β-TCP has better solubility, being replaced with newly formed bone, and has been regarded as the golden standard of bone substitutes [5]. Both ceramics, however, have poor mechanical properties, which limits their use as bone replacements.

To improve the mechanical and biological properties of β-TCP, metal dopants have been incorporated into the crystalline structure of ceramic. This is typically performed with divalent cations that replace calcium atoms in the crystal structure, such as Mg^2+^, Ag^2+^, Cu^2+^, S^r2+^, and Zn^2+^, with strontium, zinc, and magnesium being predominant [6,7]. Phosphate ions can also be replaced, for example, with SiO_4_^4+^. Doping started as a way to incorporate ions naturally occurring in the human body, such as Na, K, and Mg, to increase solubility without altering biocompatibility [5] and has moved to improve the antimicrobial [7,8], angiogenic [9], osteogenic [6,10], or mechanical properties of the implant [11,12]. It has also been shown that the substitution site relates to the material properties, and thus, different sites of substitution result in different dissolution rates, for example, affecting antimicrobial properties [13]. As an example, Hurle et al. [14] incorporated lithium ions into β-TCP brushite bone cement, which resulted in an increase in bone density. Pina et al. [15] doped the same material with Mg^2+^, Sr^2+^, and Zn^2+^. In this study, the Zn- and Sr-containing brushite cements stimulated pre-osteoblastic proliferation and osteoblastic maturation, with the presence of both zinc and strontium resulting in an even higher rate of new bone formation. Ke et al. [16] produced β-TCP doped with Sr, SiO_2_, Mg, and zinc oxides and concluded that MgO and SiO_2_ enhance osteoblastic differentiation, improving new bone formation after 16 weeks of implantation in rat femurs. Somers et al. [17] proposed β-TCP co-doped with magnesium, strontium, silver, and copper cations—Mg-Sr and Mg-Sr-Ag-Cu, when used in Direct Ink Writing, improves compressive strength and densities when compared to undoped β-TCP.

Apart from chemistry, surface topography is another important property of biomaterials, being responsible for the modulation of cellular adhesion on the material surface and subsequent cell orientation and morphology, as well as other aspects of cell activity [18]. Current trends in biomaterial engineering focus, therefore, on tailoring material surfaces to improve bone regeneration by promoting osteoblast adhesion, proliferation, and differentiation, as well as the formation of the new bone matrix (known together as osteoconductivity). Surface modifications can rely on material removal with electrochemical/chemical (acid, electrochemical solutions), mechanical methods (sandblasting), and lately, laser methods. In contrast, material deposition can also be conducted, using methods such as chemical vapor deposition (CVD), physical vapor deposition (PVD), solid-state diffusion bonding, plasma spraying, pulsed laser deposition, etc. [19,20].

Among these, laser-based techniques are the only ones that do not result in surface contamination, avoiding a reduction in biocompatibility. Laser treatments, however, may result in thermal effects and create a heat-induced secondary phase. In the case of β-TCP, this phase is transformed into α-TCP. This may result in different dissolution rates since α-TCP and β-TCP have solubility rates of 0.24 mg/L and 0.15 mg/L at 37 °C, respectively [21], inducing changes in the concentration of calcium and phosphate ions and altering cell behavior. For a correct appreciation of the effects of structuring, this phase transition should be avoided [22].

Thermal effects can be avoided by the application of ultra-short laser pulses. These pulses, due to their short duration, interact with the materials in such a way that energy is delivered only to the electrons and not to the full crystal lattice, leaving the latter unaffected by thermal effects [23,24,25]. This is of major importance when using laser ablation on materials that would otherwise suffer thermal degradation, and it is a key aspect that makes the application of ultra-short pulses for texturing metals, polymers, and ceramics possible [26]. In recent years, the application of ultra-short lasers has been extended to their use in biomaterials. For example, Götz et al. [27] used a femto-second laser to produce a laser-structured titanium alloy (Ti6Al4V) capable of improving osteointegration 12 weeks after implantation in rabbit models. On this same material, Aceti et al. developed a process for the deposition of in situ silver nanoparticle formation and nano- and micro-structuring of metal surfaces via ultra-short laser patterning, where particles are produced in aqueous solution via multiphoton photoreduction and deposited in the metal upon laser patterning, providing a green and cost-effective alternative for the functionalization of Ti surfaces and achieving a synergetic effect between the nanostructure and the antibacterial particles [24]. Sotelo et al. have also noted the generation of LIPSS on titanium and stainless steel. This structuring resulted in a reduction in bacterial viability and an enhanced osteogenic effect on both materials [28]. Carvalho et al. [29] produced topographies in alumina-toughened zirconia (ATZ) that led to an increase in human bone-derived mesenchymal stem cells using femtosecond laser ablation in a laser interference patterning setup. Some work has also been completed on the application of ultra-short laser patterning in polymer composite materials; for instance, Filipov et al. applied ultra-short laser ablation for structuring the surface of 3D-printed polycaprolactone (PCL) scaffolds. The produced patterns (microchannels and microprotrusions) resulted in an increase in surface wettability, which stimulated the proliferation of osteoblastic cells while, at the same time, having an inhibitory effect on S. aureus and E. coli within 48 h. This inhibitory effect was achieved by the disruption of S. aureus cells and the inhibition of E. coli biofilm attachment [30]. Although the versatility of the technique has been shown, studies on laser surface texturing are still, however, typically focused on metal and zirconia implants, and little has been studied on the use of this technique in TCP materials.

Our group has previously successfully used femtosecond laser irradiation to induce micropatterning on the β-TCP surface that influenced bone marrow stem cell and MC3T3 osteoblast migration while at the same time avoiding the formation of the α-TCP phase in the material [23,31]. In the current study, we investigated the production of controlled microtopographies using femtosecond laser ablation on metal-doped β-TCP for antibacterial applications. For this, an ultra-short laser system capable of producing femtosecond pulses was used to pattern ceramic disks composed of β-TCP, co-doped with several combinations of Sr^2+^, Mg^2+^, Ag^2+^, and Cu^2+,^ for improvement of phase stability and antimicrobial properties. The co-doped and undoped TCP powders were prepared through co-precipitation and then shaped into disks that were processed with the ultra-short laser system to produce different patterns on the disk’s surface. The samples produced were analyzed regarding their morphology, via SEM, optical profilometry, and confocal microscopy and regarding their chemistry via XRD and Raman spectroscopy. The values of water contact angles and surface-free energy were also obtained.

## 2. Materials and Methods

### 2.1. β-TCP Disk Production

β-TCP disks were prepared through co-precipitation in a methodology previously reported by the group [16]. In short, calcium nitrate tetrahydrate was added to distilled water in a 6 L double-walled glass reactor. Metal salts, strontium nitrate, copper (II) nitrate hemi–(pentahydrate), silver nitrate, and magnesium nitrate hexahydrate were added to the reactor when making doped β-TCP in the quantities needed to realize the stoichiometric percentages of the metals: 2% Mg, 2% Sr, 0.1% Ag, and 0.1% Cu. Afterwards, a solution of ammonium phosphate dibasic was added to the reactor using a peristaltic pump at a rate of 10 mL/min. The temperature of the reaction was kept at 31 °C and the pH at 6.7 (7.2 for all metal-doped β-TCP) with addition of ammonia. The reaction was kept under mechanical agitation, and, after the complete addition of the ammonium phosphate dibasic solution, it was left to mature for 20 h still under mechanical agitation. Afterwards, the slurry obtained was filtered and dried. The dried material was calcinated following a 3-step process and milled. The powder was later mixed with distilled water and the dispersant agent Darvan C^®^ in a ball miller, which resulted in the production of a slurry that was poured into molds, left to dry overnight at 40 °C, and finally sintered. Disks of non-doped β-TCP were produced, as well as with the following metal compositions: 2% Mg, 2% Sr, 0.1% Ag, and 0.1% Cu; 2% Mg, 2% Sr, and 0.1% Ag; 2% Mg, 2% Sr, and 0.1% Cu (denominated onwards as β-TCP@MgSrAgCu, β-TCP@MgSrAg, and β-TCP@MgSrCu, respectively) (Figure S2). An analysis of the powders before disk formation was conducted to verify the success of the process.

To verify the correct production of the β-TCP without contamination by the Ha or α-TCP phases, and to confirm the incorporation of the metals in the crystal lattice, the calcinated powders were analyzed with XRD (Figure S1) and FTIR (Figure S3). To verify the effectiveness of the milling process, laser diffraction particle size analysis was performed on the several powders produced before and after the milling process (Figure S4 and Table S1).

### 2.2. Laser Ablation

**Figure 1:**
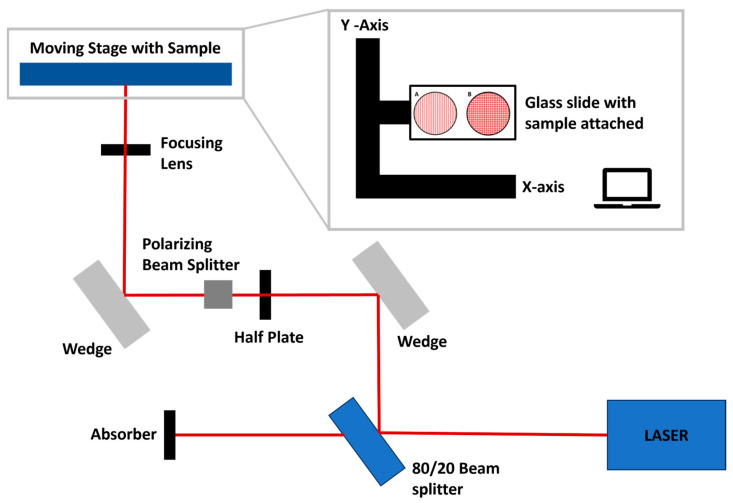
Schematic overview of laser processing setup with detail on sample pattern showing (**A**) parallel lines and (**B**) grid-like pattern.

Laser ablation was performed using a Solstice Ace system (Spectra-Physics, Milpitas, CA, USA) with a laser pulse duration of 70 fs and a central wavelength of 800 nm. For the ablation, the samples were fixed to a glass slide using double-sided tape and placed on a software-controlled (LabView) two-axis motorized translation stage (Thorlabs, Newton, NJ, USA), and the laser beam was focused on the surface using an achromatic convex lens with a 200 mm focal distance, resulting in a focal spot of 25 μm. The repetition rate was 1 kHz for all samples. The disk surface was processed with raster scanning the surface at different speeds and hatch distances (Table 1). Laser energy was adjusted using a polarization-based beam splitter and a half-wave plate. The laser ablation patterns (Figure 1) consisted of parallel lines or grid-like (crossed lines at an angle of 90°) patterns with different distances between lines (hatch distance). In the grid-like pattern, hatching distances were kept the same for horizontal and vertical lines.

### 2.3. X-ray Diffraction (XRD)

XRD was used to quantify the β-TCP, α-TCP, and HA crystalline phase percentages present in the sintered disks and confirm the incorporation of metals in the material. X-ray data were acquired (PANalytical Aeris Instrument Suite and a PIXcel1D-Medipix3 detector) between 2θ 5° and 2θ 70° at 25 °C, using a Cu-Kα1 of 1.5406 Å, 7.5 mA, a step size of 0.04 2θ°, and a scan rate of 2θ 1°/min in continuous scan mode.

For this, one sintered, non-ablated disk of each dopant combination was used for XRD to ascertain the effect of the sintering process on the composition of the material. For this, each disk was crushed manually with a mortar and alcohol until a thin powder was obtained. Afterwards, the powder was dried with N_2_ gas to make sure any residual alcohol was removed before analysis. The samples were then kept in open Eppendorf^®^ tubes in a desiccator until analysis was performed.

### 2.4. Scanning Electron Microscopy–Energy Dispersive Spectroscopy (SEM-EDS)

The surface morphology of the unprocessed samples was analyzed with SEM (Hitachi SU5000 + EDS/WDS Thermo Scientific ANAMAT). For this, the samples were covered with a thin layer of platinum prior to analysis. Images were obtained at different magnifications with accelerating voltages of 20 or 15 kV. For EDS analysis, maps of different areas of the unablated samples were analyzed at a voltage of 20 kV and a 2 k magnification in an area of 55 μm by 45 μm. Sample content in calcium, phosphate, magnesium, strontium, silver, and copper was analyzed for 3 different areas of each composition of β-TCP disks. Images of the ablated samples were obtained via SEM (Auriga Carl Zeiss Microscopy, Deutschland GmbH, Frankfurt, Germany) at an accelerating voltage of 5 kV and different magnifications, and the acquisition was conducted using a secondary electron detector. EDS mapping on ablated samples was performed at 20 kV and 1.3k magnification in an area of 89 μm by 67 μm.

### 2.5. Optical Profilometer

Optical profilometer (Leica DCM 3D, Berlin, Germany) was used to analyze ablation pattern depth and width. Images were obtained at a magnification of 20×.

### 2.6. Water Contact Angle (WCA) and Surface-Free Energy (SFE)

The wetting behavior of the investigated surfaces was assessed with solid–liquid contact angle measurements using a goniometer (KRUSS DSA100, Hamburg, Germany). To do so, a 2 μL drop of de-ionized water was used as the probe liquid in sensile drop mode. The contact angle of di-iodomethane and ethylene glycol was calculated using a 1 μL drop of the solvent as the probe liquid on the static contact angle method (Goniometer Dataphysics OCA 25). The values of the contact angle obtained for di-iodomethane and ethylene glycol (both 99% and purchased from Sigma-Aldrich, Burlington, MA, USA) were used to calculate surface-free energy, along with its polar and dispersive components, using the OWRK theory. All measurements were taken at room temperature.

### 2.7. Raman Spectroscopy

Raman spectroscopy (LabRAM HR NANO Evolution, Horiba, Japan) was used to analyze the chemical and phase composition of the synthetized β-TCP disks before and after the laser ablation processes. For this, an average of 10 accumulations was obtained between 50 and 1600 cm^−1^ with a laser wavelength of 532 nm. This technique was also used to analyze the samples after dissolution studies.

### 2.8. In Vitro Stability Studies

In vitro stability assays were made by submerging the samples in 2 mL of phosphate-buffered saline (PBS) for 48 h at 37 °C in an orbital shaker under agitation at 80 rpm. PBS was made by dissolving a PBS tablet (Sigma Aldrich, USA) in 200 mL of ultrapure water. The PBS solution was then filtered under vacuum with a 0.22 µm pore diameter sterile Mixed Cellulose Ester (MCE) filter (membrane solutions). After 48 h, the samples were rinsed with ultrapure water to remove any excess PBS and dried for 8 h at room temperature. For this assay, non-ablated samples (control) and samples ablated at 8.15 mJ/cm^2^ with a scan velocity of 0.75 mm/s and a hatch distance of 50 μm of all four dopant systems were used. After the established time, samples were analyzed with SEM and Raman spectroscopy.

### 2.9. Confocal Microscopy

Confocal microscopy was used to analyze samples of the four dopant systems ablated with laser parameters A, B, and C (Table 1). Surface area measurements were obtained, as were values of roughness for the complete analyzed surface and for the grooves produced by the laser ablation process.

## 3. Results and Discussion

### 3.1. X-ray Diffraction

**Figure 2:**
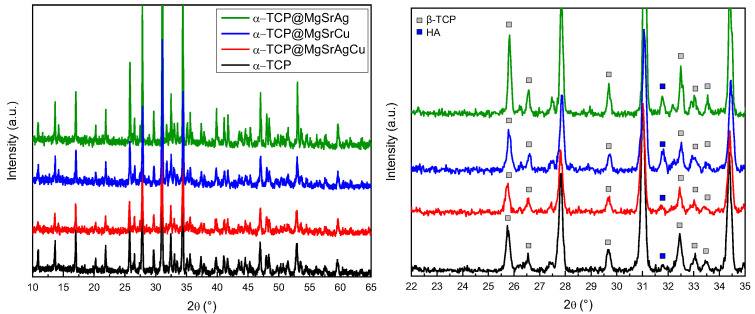
XRD spectra of the sintered β-TCP, β-TCP@MgSrAgCu, β-TCP@MgSrCu, and β-TCP@MgSrAg disks with detail on the 2θ region from 22° to 35 2θ° indicating the peaks corresponding to β-TCP, α-TCP, and HA.

XRD was used to evaluate the presence of HA [32,33] in the produced TCP powders, analyze the crystalline phase of the produced TCP, and confirm the correct incorporation of metals in the crystal lattice at Ca site 1 (Figure 2). Comparing the non-doped TCP with the doped compositions, a shift in the main peaks of the spectra suggests the substitution of the metallic ions in Ca^2+^ site 1 of TCP, as observed on the (214), (217), and (220) peaks of TCP at 2θ 28 °, 2θ 31 °, and 2θ 34.5 °, respectively. The visible peak shift to the right between doped and non-doped samples is indicative of a change in crystal lattice parameters, indicating the incorporation of different ions into the lattice leading to its deformation and consequent change in XRD pattern [34].

**Figure 3:**
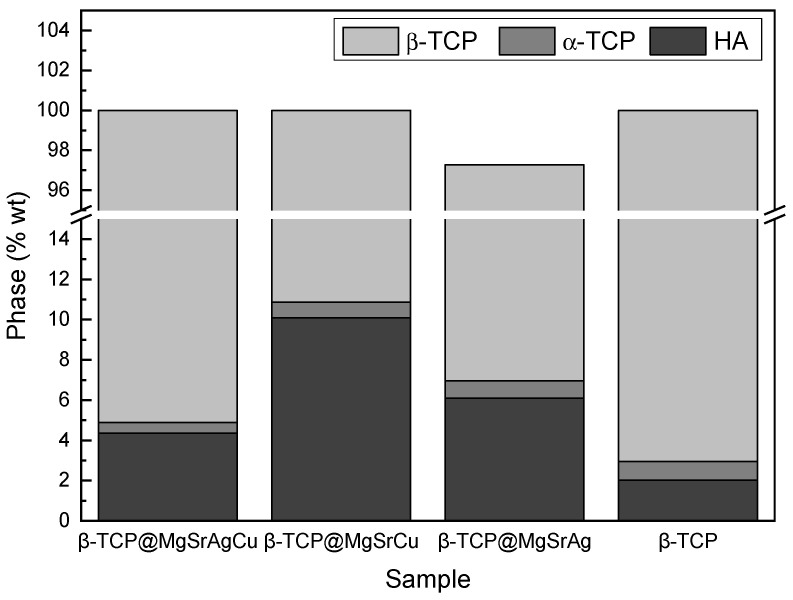
Phase composition of the sintered ceramic disks of all four dopant systems in HA, β-TCP, and α-TCP.

Phase matching was completed using the Rietveld refinement with the Profex^®^ software (Figure 3). As can be observed in the results, the percentage of α-TCP is below 1% for all produced ceramics, indicating the samples do not have a significant amount of α-TCP phase. The percentages of HA varied between samples, from 2% in β-TCP to 10% in β-TCP@MgSrCu.

### 3.2. SEM-EDS

SEM micrographs and EDS mapping were acquired for non-ablated disks of all four dopant compositions. For Ag-doped material, the surface of the disk is granular, with some cracks visible in the latter. On all other doped samples, the surface appears smooth, with some marks from the polishing process being visible on the β-TCP@SrMgAgCu sintered at 1300 °C and β-TCP@SrMgCu samples.

The EDS results (Table 2) show an excess of copper in all samples and a lack of Mg in the β-TCP@MgSrCu and β-TCP@MgSrAg samples. Overall, the incorporation of strontium occurred as desired, with the β-TCP@MgSrCu sample being the exception. It is also important to note that, due to the superimposition of the calcium and silver peaks, the latter cannot be quantified. Overall, there are no metals aside from those belonging to each composition, indicating that the samples were not contaminated in any step of the process.

**Figure 4:**
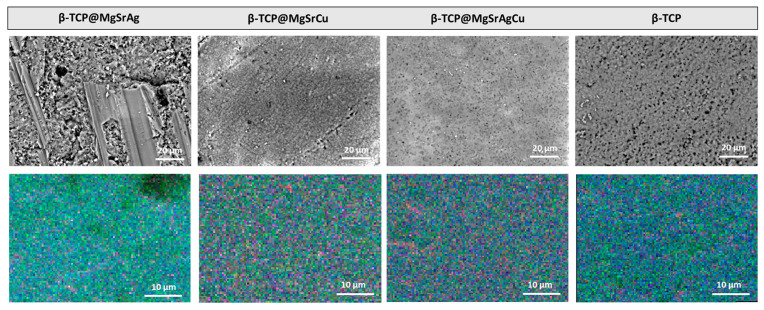
SEM micrographs and EDS mapping of unprocessed and polished samples of the four dopant systems at a magnification of 1 k and an acceleration voltage of 20 kV.

When acquiring the distribution maps of the several elements in all the β-TCP samples, using an area of 57.6 μm × 43.2 μm, at a magnification of 2 k, and at 20 kV (Figure 4), it is possible to confirm that all elements are evenly distributed in the analyzed areas, confirming that the samples are homogenous in composition. This indicates that the surface of the produced tablets would behave the same way, and there would be no particular place where a higher ion concentration or dissolution rate would be observed.

**Figure 5:**
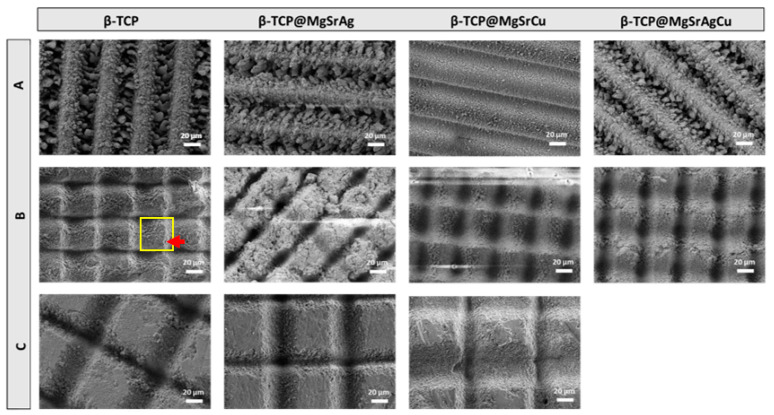
SEM micrographs obtained at an acceleration voltage of 5 kV and a magnification of 500× of β-TCP samples of the four dopant systems ablated with parameters (**A**) 8.15 mJ/cm^2^ with a scan velocity of 0.75 mm/s and a hatch distance of 50 μm, (**B**) 6.11 mJ/cm^2^ with a scan velocity of 1.5 mm/s and a hatch distance of 50 μm, and (**C**) 6.11 mJ/cm^2^ with a scan velocity of 1.5 mm/s and a hatch distance of 100 μm. A yellow square marks the ablation pattern, with a red arrow pointing out material accumulation on the non-ablated area.

Observing the SEM micrographs (Figure 5) of the samples ablated at 8.15 mJ/cm^2^ with a hatch distance of 50 μm at a scan velocity of 0.75 mm/s revealed similar topography in the surface of the ceramics with the different dopant systems. This parameter results in a large quantity of debris accumulating inside the groove and in the non-ablated area between the grooves. From the SEM images obtained, it is also possible to note that there is not a completely non-ablated area between two consecutive grooves that seem to overlap due to a short hatch distance compared to the groove width.

Upon lowering laser energy and performing the laser ablation at 6.11 mJ/cm^2^, 1.5 mm/s, and a hatch distance of 50 μm, one can observe that the area between grooves is not a flat square, as could be expected. This is due to the accumulation of ejected material from the laser ablation. The grooves produced by the ablation using parameter B are smoother when compared to parameter A, indicating that the lower energy density causes less debris to be produced upon ablation. It is also notable that the material deposition in the non-ablated area between grooves occurs more on one side than the other, as marked on the β-TCP sample ablated with this parameter, which is a result of the sample motion. Furthermore, ablating the samples with parameter B seems to produce grooves that are not completely separated by a non-ablated area, just like it happened when parameter A was used. Although the ablation pattern B consists of a grid-like pattern where vertical and horizontal grooves are ablated using the same parameters, it is clear from the images obtained that in one direction the grooves are deeper than in the other.

On the samples ablated at 6.11 mJ/cm^2^, 1.5 mm/s, and a hatch distance of 100 μm, a clear non-ablated area is observed. This area is smooth and lacks a significant accumulation of debris. Again, as with parameter B, the two directions, vertical and horizontal, do not produce similar grooves, with the grooves produced in one direction seemingly wider than those produced in the other direction.

In both grid patterns, the area where the grooves cross one another produces a deeper, conical depression in the material.

**Figure 6:**
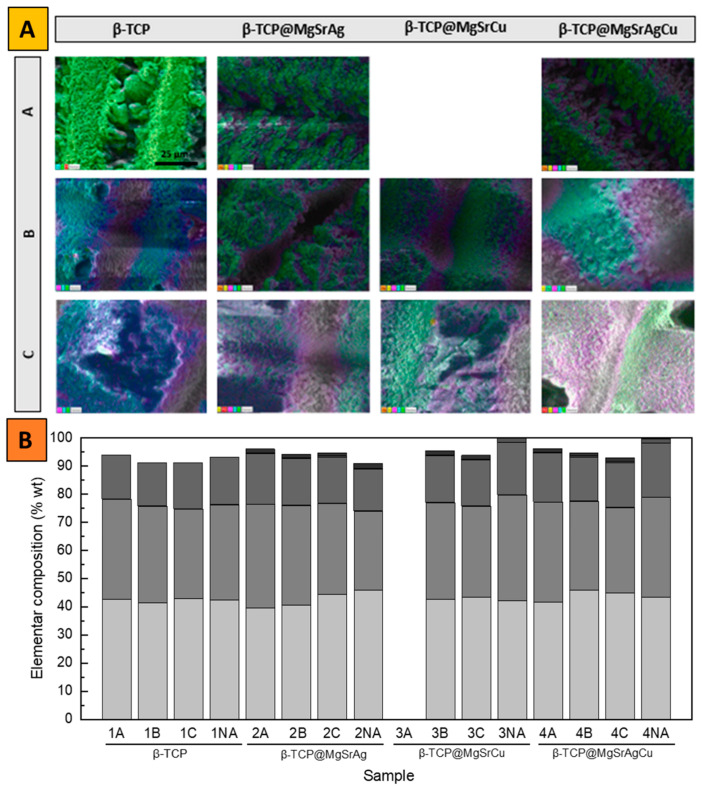
(**A**) EDS mapping of samples regarding composition on (light green) strontium, (yellow) calcium, (pink) phosphorus, (blue) magnesium, and (green) oxygen taken at an accelerating voltage of 20 kV and an amplification of 1300 times. (**B**) Elemental composition of the analyzed non-abated (NA) and samples ablated with parameters (A) 8.15 mJ/cm^2^, 0.75 mm/s, hatch distance of 50 μm, and in a line pattern; (B) 6.11 mJ/cm^2^, 1.5 mm/s, hatch distance of 50 μm, and in a grid pattern; and (C) 6.11 mJ/cm^2^, 1.5 mm/s, hatch distance of 100 μm, and in a grid pattern of all 4 dopant systems (1 to 4).

The EDS mapping indicates a homogenous surface composition. Comparing the ablated with the non-ablated areas shows that there is no significant effect of the laser processing on the composition of the samples (Figure 6A,B, respectively).

### 3.3. Optical Profilometer of Ablated TCP Disks

**Figure 7:**
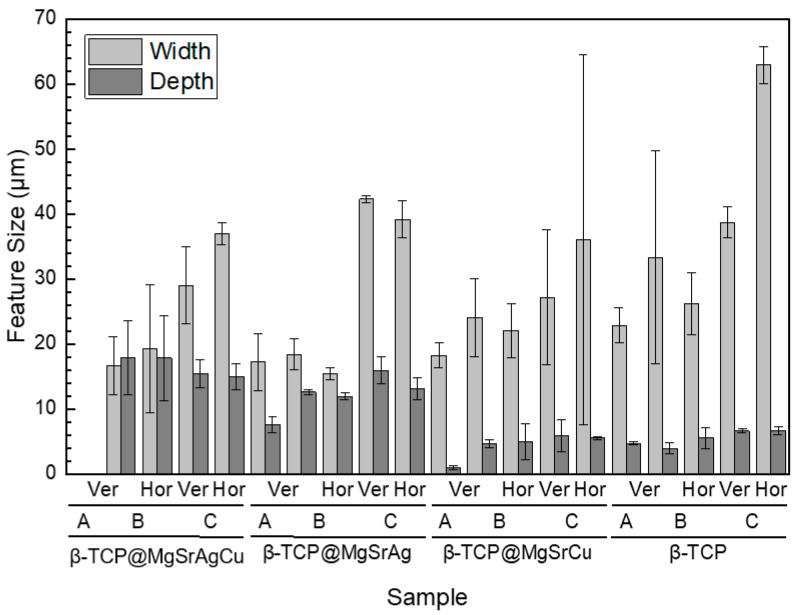
Values of groove width and depth attained for samples ablated at (A) 8.15 mJ/cm^2^, 0.75 mm/s, hatch distance of 50 μm, and in a line pattern; (B) 6.11 mJ/cm^2^, 1.5 mm/s, hatch distance of 50 μm, and in a grid pattern; and (C) 6.11 mJ/cm^2^, 1.5 mm/s, hatch distance of 100 μm, and in a grid pattern.

TCP disks were analyzed via optical profilometry to obtain the depth and width of the grooves produced for the several ablated samples (Figure 7).

In the non-doped sample and the β-TCP@MgSrCu copper-doped samples, the grooves are shallower than in the β-TCP@MgSrAgCu and the β-TCP@MgSrAg. On the β-TCP@MgSrAg and the β-TCP@MgSrCu samples with grooves with a hatch distance of 100 μm, the groove width is double when compared to the sample prepared with a hatch distance of 50 μm, which indicates an overlap of the grooves when the hatch distance is 50 μm. The groove width and depth are the same in both scanning directions when the sample is ablated in a grid pattern. Overall, the groove depth is maintained when laser energy is increased within the same sample.

These results support those from SEM since the vertical and horizontal groove widths are different in all samples, with this effect being especially noticeable on the samples ablated with parameter C.

### 3.4. Wettability and Surface-Free Energy (SFE)

All ablated samples exhibited super hydrophilic behavior, with the drop spreading completely upon contact with the surface, which inhibited the measurement of a contact angle value. For the control samples, the water contact angle (WCA) values obtained ranged between 70° and 93°, indicating that the non-ablated surfaces that have a hydrophilic behavior become superwicking surfaces after laser ablation [35].

To calculate surface-free energy (SFE), the contact angles between the surface and a drop of diiodomethane (MI) and ethylene glycol (MEG) were measured. For this purpose, 1 µL drops of each solvent was placed on the surface, and the contact angle was measured using the sessile drop method 30 s after the droplet was placed to obtain a stable contact angle value. SFE velues were calculated by the Owens, Wendt, Rabel & Kälble (WORK) model.

**Figure 8:**
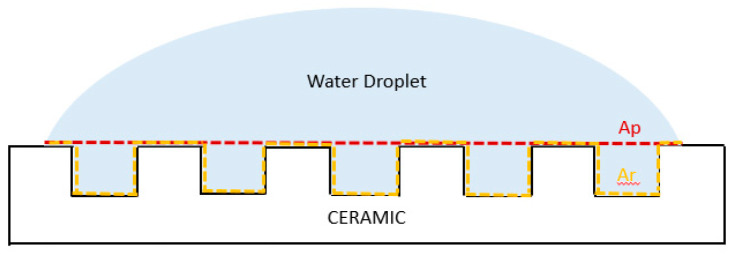
Schematic representation of a water droplet on a micro-structured ceramic surface with detail on the projected (Ap) and real (Ar) surfaces in contact with the liquid.

Similar behavior to the WCA measurements described above was observed in all ablated samples with the two tested solvents, which did not allow a value for SFE to be obtained. This increased hydrophilicity can be explained with the Wenzel model, which applies to a fully wetted surface under the droplet. This model states that a roughness factor (*f*) greater than 1 results in the real surface in contact with the liquid *Ar* being greater than the projected surface *Ap*, leading to an increase in wettability, and consequently a lower contact angle [36] (Figure 8).

This increased wettability would possibly improve protein adhesion and the healing process, as well as implant integration.

For the non-ablated samples, however, the three contact angle values, one for each solvent, were measured, and the SFE values were obtained (Table 3).

As can be seen, all WCA values are similar between the different samples, showing that the addition of dopants does not significantly affect the hydrophilicity of the surface. Furthermore, the increase in hydrophilicity is a promising property, leading to a potential improvement in cellular adhesion and ion release as well as a decrease in bacterial adhesion [37,38,39,40,41].

Regarding surface-free energy, it has been shown that it can have a significant effect on bacterial adhesion. A surface-free energy between 20 and 30 mJ/m^2^ resulted in a minimum of bacterial adhesion. Furthermore, bacterial spreading was reported to depend on the polar component of the surface-free energy, with a polar component less than 5 mJ/m^2^ reducing bacterial spreading and a polar component greater than 15 mJ/m^2^ resulting in high spreading. It can be seen in the table above that the polar component of the surface-free energy is below 1 mJ/m^2^ for all doped samples, indicating a potential reduction in bacterial spreading on the surface of the samples, even when they have not been ablated. For these samples, it is noted that the SFE values range from 20 to 35 mJ/m^2^, which corresponds to the range associated with the minimum bacterial adhesion [42].

### 3.5. Raman Spectroscopy

**Figure 9:**
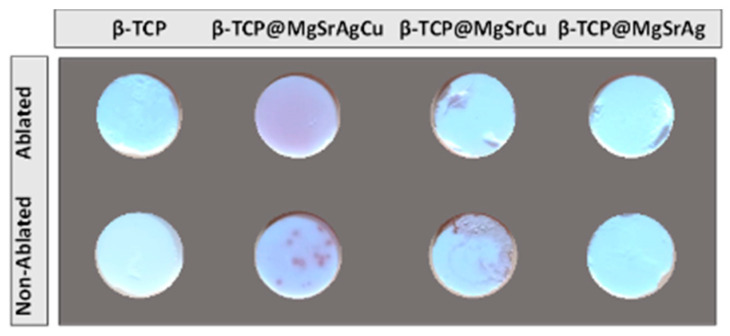
Picture of samples of all four dopant systems before and after ablation, showing the color difference induced by the process.

Due to a change in color from white to pink in copper-containing disks (Figure 9) and the intensification of this color after laser ablation, it was hypothesized that sintering and laser ablation led to the formation of copper oxides in the samples.

**Figure 10:**
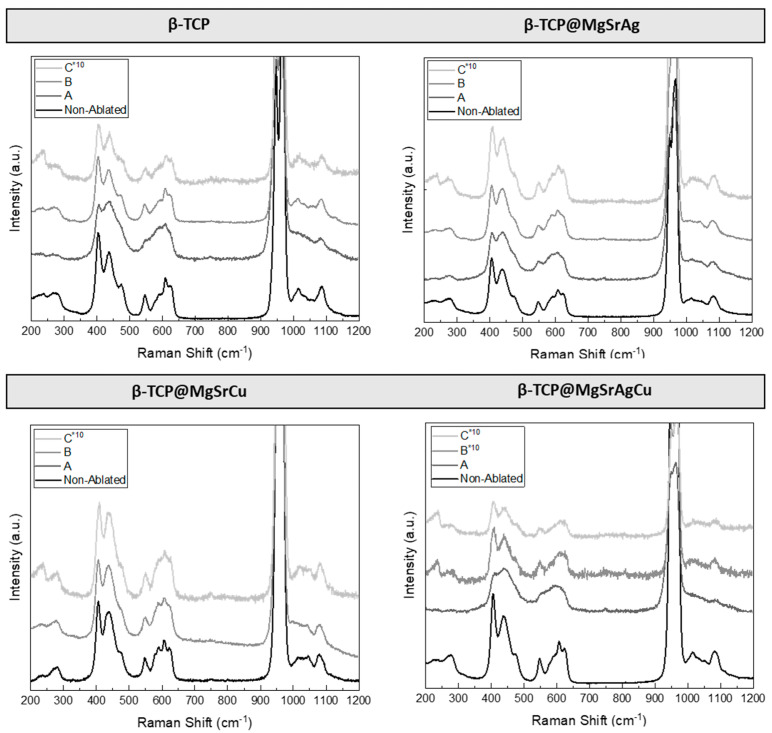
Raman spectra obtained for doped and non-doped β-TCP disks at (A) 8.15 mJ/cm^2^, 0.75 mm/s, hatch distance of 50 μm and in a line pattern, (B) 6.11 mJ/cm^2^, 1.5 mm/s, hatch distance of 50 μm and in a grid pattern, and (C) 6.11 mJ/cm^2^, 1.5 mm/s, hatch distance of 100 μm and in a grid pattern. Note that for spectra marked with *10, the intensity values were multiplied by 10 to facilitate peak visualization.

To evaluate the veracity of this theory, Raman analysis was conducted on ablated β-TCP disks of the four different compositions at the several ablation parameters used (Figure 10). The spectra of the ablated sample were compared to those of the non-ablated sample for each composition.

On the Raman spectra, the bands are due to the vibrations of the phosphate groups. The “free” tetrahedron PO_4_ possesses four normal modes of vibration, decomposed as 1*A_1_* (ν_1_) + 1*E* (ν_2_) + 2*T*_2_ (ν_3_ and ν_4_). *E* and *T*_2_ are doubly and triply degenerate, respectively, and *A*_1_ is triple degenerated, consisting of two peaks and a shoulder. The bands at 920–1000 cm^−1^ are assigned to the ν_1_ vibrations. The band related to P(1)O_4_ is observed at ~950 cm^−1^. The P(2)O_4_ and P(3)O_4_ give the bands at ~956 and ~970 cm^−1^, respectively. The vibrational features are sensitive to the site of substitution: P(3) is sensitive to Ca(5) substitution and to the valence of the cation; P(1) is sensitive to monovalent substitution on Ca(4) and to the nature of the cation on Ca(4) [43].

In addition to the υ1 mode, triple degenerate bands corresponding to O-P-O bending (υ4) and doubly degenerate bands corresponding to E symmetry of the O-P-O bond (υ2) are observed in the regions of 570–625 cm^−1^ and 400–490 cm^−1^, respectively. The υ2 and υ4 modes appear very close together and in a broad range in the β-TCP spectrum [44]. The ν3 frequency (1020 cm^−1^–1095 cm^−1^) arises from the triply degenerate T2 mode involving asymmetric PO stretching and also P motion. In addition to the internal P0_4_^3−^ bands, several peaks are detected between 50 and 320 cm^−1^, which are due to the translational modes of the Ca^2+^ and P0_4_^3−^ sublattices [44].

There are three copper oxide and hydroxide compounds that are known to be stable as bulk solids at ambient temperatures. These are Cu_2_O (cuprous oxide), CuO (cupric oxide), and Cu(OH) (cupric hydroxide). Cu_2_O is known to have bands around 415 cm^−1^, 525 cm^−1^, and 625 cm^−1^. CuO presents a high-intensity peak at 288 cm^−1^ and one low-intensity band at 337 cm^−1^. For Cu(OH)_2_, the Raman bands at 292 cm^−1^ and 483 cm^−1^ have been reported [45]. As can be seen, the bands for copper II oxide (CuO) and copper I oxide (Cu_2_O) overlap β-TCP peaks, making it impossible to isolate either compound in the spectra. Furthermore, it is also noted in the Raman spectra that the laser ablation did not cause thermal degradation with the resultant transformation of the β phase to the α phase of the calcium phosphate.

### 3.6. In Vitro Bioactivity

**Figure 11:**
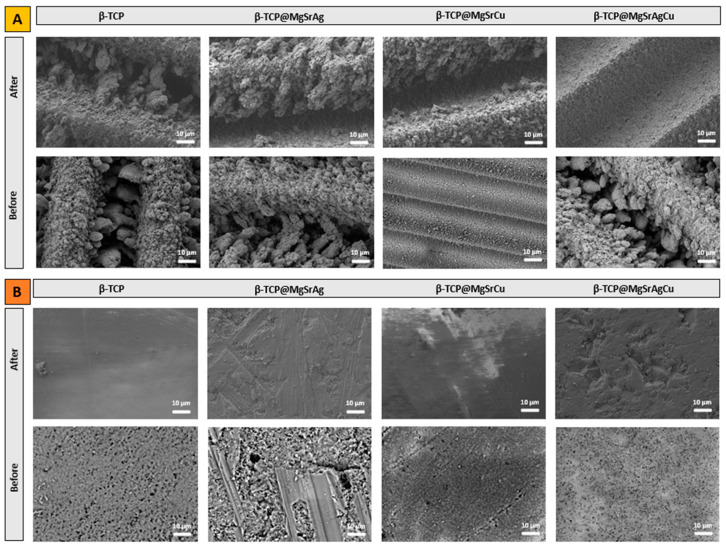
SEM micrographs of (**A**) ablated and (**B**) non-ablated samples of the four dopant systems before and after the bioactivity assays taken at an amplification of 500× and at an acceleration voltage of 5 kV, except images of ablated β-TCP@MgSrCu before bioactivity assays and non-ablated samples before bioactivity assays, which were taken at an acceleration voltage of 15 kV and 20 kV, respectively.

Bioactivity assays were performed, and SEM images were obtained of the sample surface after 48 h of submersion in PBS (Figure 11). These images are compared to those obtained for non-submerged samples to investigate the stability of the samples, concerning their dissolution in PBS, and any possible change in morphology upon submersion in PBS. As can be seen, the morphology of samples submerged and non-submerged is similar, with deposited material still visible even after 48 h of submersion in PBS. These results indicate that the samples are stable within this period; thus, any antibacterial or osteoinductive effect induced by the surface topography would be maintained in the first 48 h.

**Figure 12:**
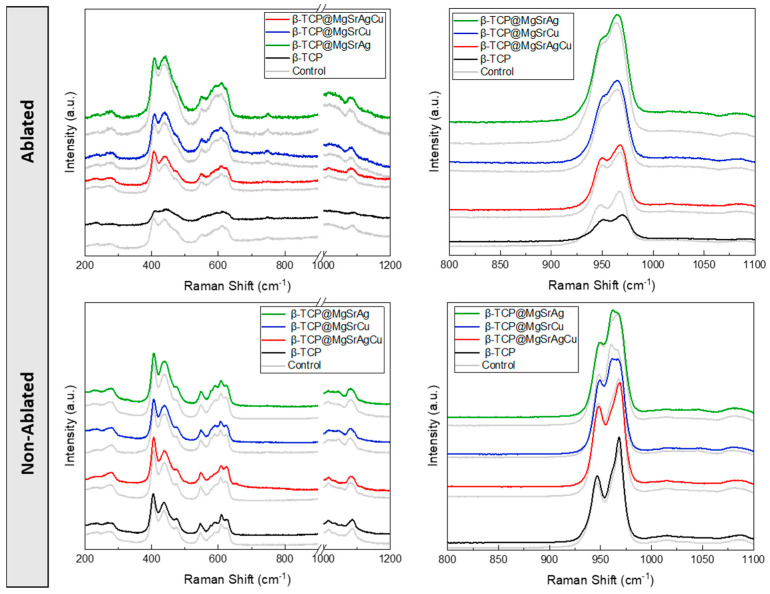
Raman spectra obtained for (black) β-TCP, (red) β-TCP@MgSrAgCu, (blue) β-TCP@MgSrCu, and (green) β-TCP@MgSrAg disks ablated at 8.15 mJ/cm^2^, 0.75 mm/s, hatch distance of 50 μm, and in a line pattern before (control) and after submersion in PBS for 48 h.

Raman spectroscopy was used to evaluate if the submersion in PBS led to any changes in the chemical and phase composition of the TCP disks. The spectra obtained were compared to those of the same samples that were not submerged in PBS (Figure 12).

The Raman spectra do not show any major differences between the control and submerged samples.

The doubly degenerate bands corresponding to the υ2 mode of the O-P-O bond 400–490 cm^−1^ are present, with the two peaks centered at 410 cm^−1^ and 440 cm^−1^. The triple degenerate bands of the υ4 mode of O-P-O bending in the region of 570–625 cm^−1^ are present, with peaks centered at around 580 cm^−1^, 595 cm^−1^, and 610 cm^−1^. The triply degenerate bands due to the ν_1_ vibrations of the phosphate groups at 920–1000 cm^−1^ present as a doubly degenerate band with a peak centered at 950 cm^−1^ and another centered at 967 cm^−1^. The triply degenerate ν3 frequency, from 1020 cm^−1^ to 1095 cm^−1^ from asymmetric PO and P motion, is present as a single broad band centered at 1080 cm^−1^ [43,44,46].

Raman results indicate the samples are chemically stable within the used time frame, which would suggest that the behavior of both bacterial and host cells would not drastically change within the first 48 h. This, together with the SEM results, shows that the laser ablation process does not compromise, mechanically or chemically, for the material, which remains stable even in conditions mimicking the implantation site.

### 3.7. Confocal Microscopy

**Figure 13:**
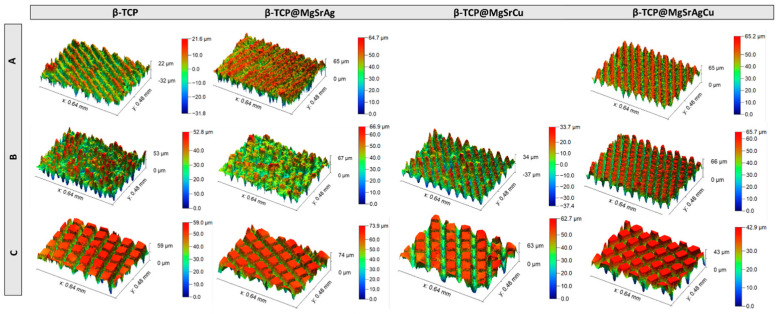
Confocal microscope 3D images of the samples of the four dopant systems ablated with parameters (**A**) 8.15 mJ/cm^2^ with a scan velocity of 0.75 mm/s and a hatch distance of 50 μm; (**B**) 6.11 mJ/cm^2^ with a scan velocity of 1.5 mm/s and a hatch distance of 50 μm; and (**C**) 6.11 mJ/cm^2^ with a scan velocity of 1.5 mm/s and a hatch distance of 100 μm.

Confocal microscopy was used to obtain 3D images of the samples of the four dopant systems ablated with all three parameters A, B, and C (Figure 13), as well as the surface area and specific surface area of these samples. Looking at the samples ablated with parameter A, one can see that the depth and morphology of the grooves produced vary, both between samples and within the same sample, with the β-TCP@MgSrAgCu sample presenting the most homogenous ablation pattern. Samples ablated with parameter B are noticeably different from one another, with, as it happened with parameter A, the β-TCP@MgSrAgCu sample presenting the most homogenous ablation pattern and the β-TCP@MgSrAg sample the least homogenous one. This last sample is also the one that presents the shallowest grooves. Parameter C results in the most homogenous surfaces, with no particular sample standing out.

Overall, laser ablation led to an increased specific surface area (Table 4). This, combined with increased wettability, potentiates ion release from the samples, improving their antibacterial and osteogenic induction properties [38,39].

Apart from specific surface areas, confocal microscopy was also used to obtain the roughness values of the several ablated samples (Table 5). These values were obtained for the overall surface (_T_Sa), which was compared to that of the control samples (_C_Sa), and the roughness of the produced groove (_G_Sa). All values were obtained as Sa (arithmetic mean height) ± Sq (squared mean height).

Regarding the total roughness of the sample surfaces, in general, all samples show a roughness increase upon laser ablation for all three tested parameters. Samples of β-TCP@MgSrAgCu ablated with parameters B and C are the exception, being that the first produced similar roughness to that of the control and the second resulted in an 85% roughness decrease. It is also notable that all samples show micro- or submicro-total roughness, ranging from 0.13 μm to 16 μm. These values also reveal that there is no clear relationship between the roughness values and either laser energy or hatch distance.

Concerning the roughness of the produced grooves, the values obtained are not significantly different from those of the control surface, with, once again, the exception of β-TCP@MgSrAgCu ablated with parameters B and C, which show a roughness increase of 16 and 8 times the value of the control, respectively. Furthermore, these results reveal that any surface structuring of the ablated areas is of micro- or submicron-scale, which is favorable for the improvement of antibacterial properties, namely via the inhibition of bacterial biofilm attachment [47,48].

## 4. Conclusions

Pure and metal-doped β-TCP disks were produced, and the XRD results, supported by EDS analysis, confirm the incorporation of the metals into the crystal lattice. Several laser parameters were used to ablate the surface of the produced disks in line and grid patterns, and SEM analysis confirmed the successful surface ablation in the various desired patterns. Raman spectroscopy and EDS analysis of the ablated disks prove that laser processing does not induce significant changes in the elemental or chemical composition of the ceramics, confirming the suitability of the technique and chosen parameters for the modification of β-TCP. Confocal microscopy shows that laser ablation increases roughness and specific surface area, with these values being lower and more constant at larger hatch distances. This, in combination with WCA measurements, which reflect the super hydrophilic behavior of all ablated samples, reflects the potential improvement of the ion release mechanism in the ceramics by increasing ion diffusion. Furthermore, both wettability and roughness results indicate the potential to reduce bacterial spreading/adhesion, thus improving antibacterial activity, although protein and cellular adhesion studies should be performed to confirm this possibility.

Overall, we have shown the possibility of using femtosecond lasers for surface patterning of β-TCP and β-TCP doped with magnesium, strontium, and silver (β-TCP@MgSrAg); magnesium, strontium, and copper (β-TCP@MgSrCu); and magnesium, strontium, silver, and copper (β-TCP@MgSrAgCu). The micro/structuring of these materials leads to improved wettability without detrimental effects. Although further studies are necessary to verify the bacterial and osteoblastic responses to the produced textured β-TCP, our results are promising for the use of femtosecond laser ablation of TCP as a method to improve the osteogenic and antibacterial properties of β-TCP.

## Figures and Tables

**Table 1:** Laser ablation parameters used to depict chosen scanning velocity (V), laser fluence (F), hatch distance (H), and ablation pattern.

Designation	V (mm/s)	F (J/cm^2^)	H (μm)	Pattern
Parameter A	0.75	8.15	50	Lines
Parameter B	1.5	6.11	50	Grid
Parameter C	1.5	6.11	100	Grid

**Table 2:** Atomic percentages of strontium (Sr), magnesium (Mg), copper (Cu), and silver (Ag), with corresponding percentages of the theoretical value between brackets, as well as the ratio between calcium and phosphate (Ca/P) obtained via EDS mapping for all produced β-TCP formulas.

Sample	Sr (%)	Mg (%)	Cu (%)	Ag (%)	Ca/P
Theoretical value	2	2	0.1	0.1	1.5
Non-Doped	-	-	-	-	1.60
β-TCP@MgSrAgCu	1.95 (97.5)	1.85 (92.5)	0.14 (140)	-	1.50
β-TCP@MgSrCu	1.61 (80.5)	0.65 (32.5)	0.17 (170)	-	1.19
β-TCP@MgSrAg	1.91 (95.5)	1.00 (50.0)	-	-	1.56

**Table 3:** Surface-free energy (SFE) values, with their polar (Pol) and dispersive (Dis) components, obtained for the ablated and non-ablated ceramic disks, and contact angle values obtained for the several probes: (MI) di-iodomethane, (MEG) ethylene glycol, and (H_2_0) distilled water.

	Solvent Contact Angle (°)			SFE (mJ/m^2^)		
Sample	MI	MEG	H_2_O	Total	Dis	Pol
β-TCP@MgSrAgCu	51.5 ± 1.00	51.2 ± 8.25	92.8 ± 11.4	34.31	33.43	0.080
β-TCP@MgSrCu	60.3 ± 3.19	85.7 ± 2.00	70.6 ± 5.02	27.8	27.12	0.65
β-TCP@MgSrAg	54.1 ± 6.04	65.6 ± 14.6	69.2 ± 0.18	30.86	30.78	0.08
β-TCP	52.1 ± 0.82	61.8 ± 2.33	82.1 ± 10.1	73.45	16.8	56.64

***MI: ****Di-iodomethane; **MEG: ** Ethylene Glycol; **H_2_O: ** Water; **Dis: ** Dispersive; and **Pol: ** Polar*.

**Table 4:** Average surface area (SA) with respective standard error and specific surface area (SSA) obtained for samples of all four dopant systems non-ablated (NA) and ablated with parameters (A) 8.15 mJ/cm^2^ with a scan velocity of 0.75 mm/s and a hatch distance of 50 μm, (B) 6.11 mJ/cm^2^ with a scan velocity of 1.5 mm/s and a hatch distance of 50 μm, and (C) 6.11 mJ/cm^2^ with a scan velocity of 1.5 mm/s and a hatch distance of 100 μm.

Parameter	Sample	SA (mm^2^)	SSA (m^−1^)
A	β-TCP	0.46 ± 0.01	1.51
	β-TCP@MgSrAg	0.56 ± 0.03	1.85
	β-TCP@MgSrCu		
	β-TCP@MgSrAgCu	0.55 ± 0.00	1.80
B	β-TCP	0.55 ± 0.01	1.80
	β-TCP@MgSrAg	0.47 ± 0.01	1.54
	β-TCP@MgSrCu	0.59 ± 0.00	1.95
	β-TCP@MgSrAgCu	0.65 ± 0.00	2.13
C	β-TCP	0.48 ± 0.01	1.57
	β-TCP@MgSrAg	0.48 ± 0.01	1.59
	β-TCP@MgSrCu	0.47 ± 0.00	1.54
	β-TCP@MgSrAgCu	0.45 ± 0.00	1.48

**Table 5:** Values of area roughness obtained for samples ablated at (A) 8.15 mJ/cm^2^, 0.75 mm/s, hatch distance of 50 μm, and in a line pattern; (B) 6.11 mJ/cm^2^, 1.5 mm/s, hatch distance of 50 μm, and in a grid pattern; and (C) 6.11 mJ/cm^2^, 1.5 mm/s, hatch distance of 100 μm, and in a grid pattern showing the whole surface roughness (_T_Sa), the roughness of the produced groove (_G_Sa), and the roughness of the control sample (_c_Sa).

	_T_Sa ± _T_Sq (μm)			_G_Sa ± _G_Sq (μm)			_C_Sa ± cSq (μm)
Sample	A	B	C	A	B	C	NA
β-TCP	5.7 ± 7.0	8.4 ± 11	16 ± 19	1.7 ± 2.1	2.9 ± 3.4	1.3 ± 1.9	1.8 ± 2.4
β-TCP@MgSrAg	16 ± 19	6.8 ± 8.6	10 ± 12	2.7 ± 3.3	0.8 ± 1.1	0.99 ± 1.3	1.1 ± 1.6
β-TCP@MgSrCu		15 ± 18	14 ± 17		1.6 ± 1.8	0.17 ± 0.24	0.5 ± 1.1
β-TCP@MgSrAgCu	7.2 ± 8.4	1.0 ± 1.2	0.13 ± 0.18	0.47 ± 6.0	16 ± 19	8.5 ± 9.9	0.9 ± 0.3

## Data Availability

Data is contained within the article or supplementary material.

## Supplementary Materials

The following supporting information can be downloaded at https://www.mdpi.com/article/10.3390/bioengineering10121392/s1, Figure S1: XRD spectra of the obtained TCP powders with detail on the 2θ region from 22 ° to 35 ° indicating the peaks corresponding to β-TCP (grey), α-TCP (red), and HA (blue); Figure S2: Phase composition of the analyzed calcinated ceramic disks of all four dopant systems in HA, β-TCP, and α-TCP, with results presented as an average of the several taken measurements; Figure S3: FTIR spectra of the several produced powders, with grey lines indicating the peaks for pyrophosphate (CPP) at 727 cm-1 and 1200 cm^−1^; Figure S4: DLS of the obtained powders before (left) and after (right) 4 h of milling in a ball miller at 100 rpm; Table S1: average diameter and surface area of the several produced powders before and after milling.
