# Supplementary material for: Improvement of Metal-Doped β-TCP Scaffolds for Active Bone Substitutes via Ultra-Short Laser Structuring
Source: Bioengineering (Basel). 2023 Dec 6;10(12):1392. doi: 10.3390/bioengineering10121392 (PMC10741177; doi:10.3390/bioengineering10121392)
Supplement: Supplementary file 1 [file bioengineering-10-01392-s001.zip › bioengineering-2616712-supplementary.pdf]

Supplementary Materials

X-Ray diffraction

X-ray diffraction (Malvern Panalytical XPERT-PRO) was used to analyze the crystalline phase present in the synthetized β-TCP powders, namely the percentages of β-TCP, α-TCP, and HA and confirm the correct incorporation of metals in the crystal lattice, in the Ca site 1. For this, data was obtained between 2θ 10 ° and 2θ 80 ° at 25 °C, using a Cu-Kα1 of 1.5406 Å, 40 mA, a step size of 2θ 0.01 °, and an integration time of 80 s in continuous scan mode (**Figure S1**).

Comparing the non-doped TCP with the doped compositions, a shift in the main peaks of the spectra suggests the substitution of the metallic ions in Ca^{2+} site 1 of TCP, as observed on the (214), (217), and (220) peaks of TCP at 2θ 28°, 31 ° and 34 ° respectively. The visible peak shift to the right between doped and non-doped samples is indicative of a change in crystal lattice parameters, indicating the incorporation of different ions into the lattice leading to its deformation and consequent change in XRD pattern[34].

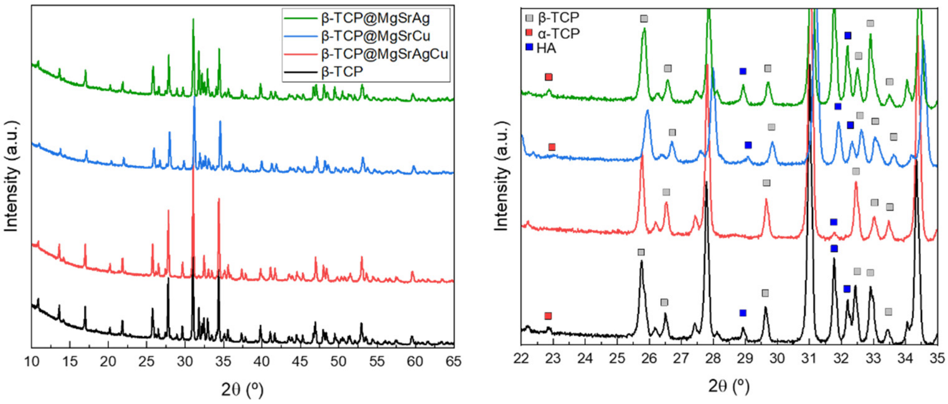

Figure S1. XRD spectra of the obtained TCP powders with detail on the 2θ region from 22 ° to 35 ° indicating the peaks corresponding to β-TCP (grey), α-TCP (red), and HA (blue).

Phase matching was done using the Rietveld refinement using Profex® software (**Figure S2**). As can be observed in the results, there is no presence of α-TCP in any of the produced ceramics, indicating the lack of thermal degradation of these samples upon calcination. Furthermore, all spectra indicate the presence of HA[32,33], this percentage is of : 1.7 % to 9.3 % in the β-TCP@MgSrAgCu sample, 33 % to 66 % in the β-TCP@MgSrAg sample, 6.0 % to 12 % in the β-TCP@MgSrCu sample, and 13 % to 17 % in the non-doped β-TCP sample

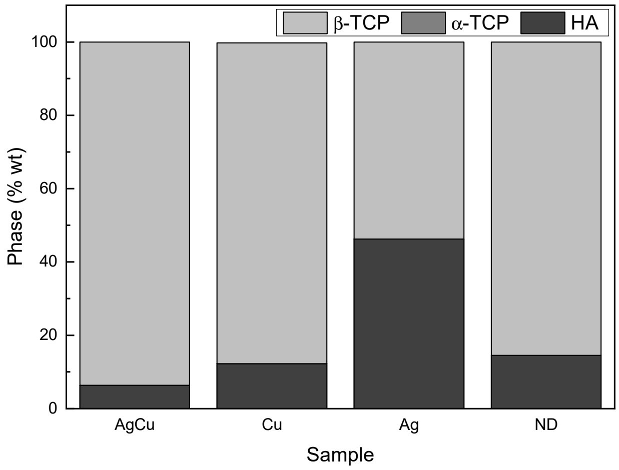

Figure S2. Phase composition of the analyzed calcinated ceramic disks of all four dopant systems in HA, β-TCP, and α-TCP, with results presented as an average of the several taken measurements.

Fourrier-Transformed Infra-Red Spectroscopy (FTIR)

FTIR (Jasco FT-IR 4600) was used on synthetized β-TCP powders to confirm the absence of calcium pyrophosphate (CPP). For this, spectra were measured at room temperature between 400-4000 cm^{-1}, with a step of 4 cm^{-1} (**Figure S3**).

As can be noted from the obtained spectra, the bands of the vibrational modes of $–PO^4$ groups appear at 493, 563, 601, 1025, 1103, and 1133 cm^{-1} and those of –OH groups of apatite phase at 624 cm^{-1}. The band at 867 cm^{-1} corresponds to residual nitrates and ammonia (NO^{3-}, NH^{4+}) derived from the reagents used in the synthesis of powders.

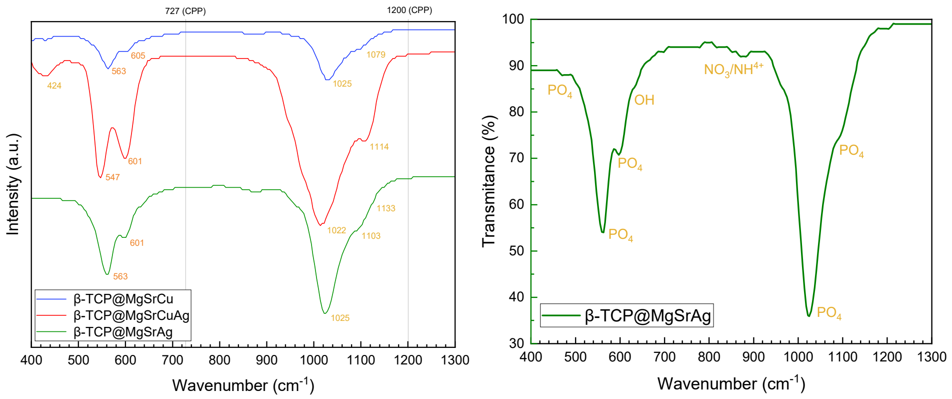

Figure S3. FTIR spectra of the several produced powders, with grey lines indicating the peaks for pyrophosphate (CPP) at 727 cm^{-1} and 1200 cm^{-1}.

No bands of pyrophosphate are visible in any of the synthetized. Furthermore, the double degenerate band at 540 cm^{-1} to 620 cm^{-1} indicates the presence of β-TCP phase.

Laser diffraction particle size analysis

Laser diffraction particle size analysis (Mastersizer3000) was used to measure average particle diameter before and after the milling process, to assess its efficacy. For these measures, distilled water was used as dispersant, and particle refraction and absorption indexes were set as those of TCP, at 1.630 and 0.1 respectively. The diffusion was modelled with the Mie theory, assuming non-spherical particles. An average of 10 measures, with an interval of 10 s between them was taken and obscuration kept between 5 % and 10 %.

From the results obtained (**Figure S4** and **Table S1**), it is possible to conclude that the milling process was successful, with the average diameter of the powders decreasing by approximately 30 % for all doped samples and 16 % for the non-doped TCP. This decrease in particle size is joined by an increase of particle surface area, being the increase of 319 %, 206 %, 196 %, and 324 % for β-TCP@MgSrCuAg, β-TCP@MgSrCu, β-TCP@MgSrAg and non-doped β-TCP respectively.

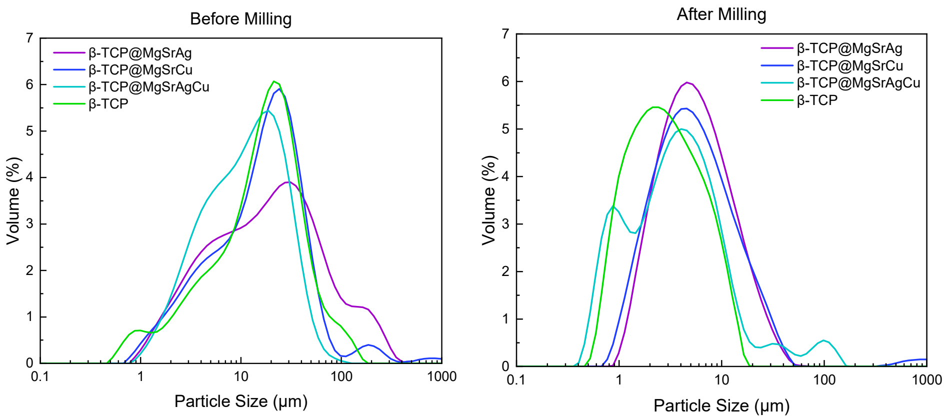

Figure S4. DLS of the obtained powders before (left) and after (right) 4 h of milling in a ball miller at 100 rpm.

Table S1. average diameter and surface area of the several produced powders before and after milling.

	**Milled**				**Non-Milled**			
	**S_o (m^2/Kg)**	**Diameter (µm)**			**S_o (m^2/Kg)**	**Diameter (µm)**		
**Sample**		D_{10}	D_{50}	D_{90}		D_{10}	D_{50}	D_{90}
β-TCP@MgSrAgCu	2643	0.886	3.72	13.1	828.7	3.10	12.2	34.0
β-TCP@MgSrCu	1488	1.84	5.42	19.7	721.6	3.24	18.2	50.2
β-TCP@MgSrAg	1362	2.12	5.63	17.2	695.4	3.19	19.9	102
β-TCP	2690	1.05	2.88	8.87	831.3	3.06	18.0	49.7
